# Leucine Supplementation in Middle-Aged Male Mice Improved Aging-Induced Vascular Remodeling and Dysfunction via Activating the Sirt1-Foxo1 Axis

**DOI:** 10.3390/nu14183856

**Published:** 2022-09-17

**Authors:** Zhujing Hao, Guiwen Xu, Mengyang Yuan, Ruopeng Tan, Yunlong Xia, Yang Liu, Xiaomeng Yin

**Affiliations:** 1 Institute of Cardiovascular Diseases, The First Affiliated Hospital of Dalian Medical University, Dalian 116014, China; 2 Department of Cardiology, The First Affiliated Hospital of Dalian Medical University, Dalian 116014, China

**Keywords:** aging, vascular remodeling, leucine, metabolism

## Abstract

Vascular aging is associated with metabolic remodeling, and most studies focused on fatty acid and glucose metabolism. Based on our metabolomic data, leucine was significantly reduced in the aortas of aged mice. Whether leucine supplementation can reverse aging-induced vascular remodeling remains unknown. To investigate the effectiveness of leucine, male mice at 15 or 18 months were supplemented with leucine (1.5%) for 3 months. All the aged mice, with or without leucine, were sacrificed at 21 months. Blood pressure and vascular relaxation were measured. H&E, Masson’s trichrome, and Elastica van Gieson staining were used to assess aortic morphology. Vascular inflammation, reactive oxidative stress (ROS), and vascular smooth muscle cell (VSMC) phenotype were also measured in mouse aortas. Compared with the 21-month-old mice without leucine, leucine supplementation from 15 months significantly improved vascular relaxation, maintained the contractile phenotype of VSMCs, and repressed vascular inflammation and ROS levels. These benefits were not observed in the mice supplemented with leucine starting from 18 months, which was likely due to the reduction in leucine transporters Slc3a2 or Slc7a5 at 18 months. Furthermore, we found benefits from leucine via activating the Sirt1-induced Foxo1 deacetylation. Our findings indicated that leucine supplementation in middle-aged mice improved aging-induced vascular remodeling and dysfunction.

## 1. Introduction

Advanced age has been recognized as an independent risk factor for cardiovascular and cerebrovascular diseases [1]. Aging-induced vascular remodeling, which is characterized by increased inflammatory responses [2,3], enhanced oxidant stress [4], and vascular smooth muscle cell (VSMC) phenotype transition [5], leads to wall thickening and lumen enlargement [6]. Therefore, an urgent need arises to clarify the mechanisms underlying the vascular aging process and further develop new preventive approaches.

Metabolism remodeling occurs during aging, and the disruption of metabolic homeostasis accelerates aging [7]. Metabolism studies on vascular aging over the past few decades are mainly focused on fatty acid [8] and glucose utilization [9], and little is known about the metabolic changes in amino acids and their effects on aging-induced vascular remodeling and dysfunction. Amino acids regulate cell function not only as nutrients but also as direct contributors to the cell signaling pathways [10]. Whether the changes in amino acid concentration in the aged aorta contribute to vascular structural and functional remodeling has not been reported.

Leucine promotes human skeletal muscle anabolism through its activation of the mechanistic target of rapamycin complex 1 (mTORC1) signaling [11,12]. In one study, exercise combined with leucine supplementation improved protein synthesis and the contractile function of skeletal muscle cells in aged mice [13]. Furthermore, several studies have shown that leucine is involved in lipid metabolism [14,15], and leucine supplementation effectively attenuates atherosclerosis in apoE knockout mice by improving the plasma lipid profile and reducing systemic inflammation [16]. Leucine is an essential amino acid and cannot be synthesized within the body. To date, no available dietary or nutritional interventions have been reported to prevent the vascular aging process and ameliorate age-related vascular remodeling. Thus, whether leucine supplementation can reverse aging-induced vascular remodeling deserves further study.

In the present study, we profiled the metabolism characteristics of the aged aorta and identified leucine as an important metabolite during vascular aging. Additionally, we sought to explore the therapeutic effectiveness and mechanisms of leucine supplementation in aging-induced vascular remodeling and dysfunction, providing a novel intervention for age-related vascular diseases.

## 2. Methods

### 2.1. Experimental Animals and Diet

C57BL/6J male mice were purchased from the Animal Center of Vital Rivers (Beijing, China). The mice were maintained at 22 °C on a 12 h light–dark cycle and given free access to water and a normal diet before the experiment. All the mice were weighed and randomly assigned to one of four dietary groups (6 mice per group). Two-month-old (2M) mice fed with a normal diet were used as the control group, and 21-month-old mice (21M) fed with a normal diet were used as the aging group. The mice that were supplemented with 1.5% leucine (L8000, Sigma Aldrich, St. Louis, MO, USA) in drinking water from 15 to 18 months and then changed to a normal diet from 18 to 21 months [16,17,18] were defined as 15M + leucine. The mice that were supplemented with 1.5% leucine in drinking water during 18 to 21 months were defined as 18M + leucine. The mice that received leucine supplementation were treated with the Foxo1 inhibitor AS1842856 (20 mg/kg, HY-100596, MCE) intraperitoneally from 15 to 18 months and were defined as the 15M + leucine + AS1842856 group. All the mice that received leucine were sacrificed at 21 months. Body weight and food intake were routinely recorded on a weekly basis.

### 2.2. Measurement of Systolic Blood Pressure

Systolic blood pressure (SBP) was measured by a tail-cuff system (BP-98A, Softron, Tokyo, Japan). During the measurement, 10 individual readings were recorded, and the average of readings was used.

### 2.3. Vascular Relaxation Assessment

Aortas were gently isolated from the mice, and perivascular fat was stripped and then cut into 4 mm segments. The aortic rings were gently mounted on force transducers, and the organ chambers were filled with a Krebs solution (in mM: 110.8 NaCl, 5.9 KCl, 25.0 NaHCO_3_, 1.07 MgSO_4_, 2.49 CaCl2, 2.33 NaH_2_PO_4_, and 11.51 glucose, pH 7.4). We stretched the aorta to 3.5 mN as initial tension, and after 1 h stabilization, phenylephrine was added to the chamber for precontraction. The vascular responses to the increasing concentrations of acetylcholine (ACh) (1 × 10^−9^ to 1 × 10^−4^ mol/L) and sodium nitroprusside (SNP) (1 × 10^−9^ to 1 × 10^−5^ mol/L) were recorded. The mechanical activity was recorded by using a force transducer (DMT, Multi Wire Myography System-620M, Aarhus, Denmark) connected to a recorder (DMT, Multi Wire Myography System-620M, Aarhus, Denmark).

### 2.4. Untargeted Metabolomic Analysis and Data Processing

The aortic specimens from mice at the age of 2M and 21 MI were ground into a powder with liquid nitrogen; then, a 400 μL methanol/acetonitrile/water solution (4:4:2, *v*/*v*) was added and mixed by vortex, stored at −20 °C for 60 min, and centrifuged at 14,000× *g* for 20 min at 4 °C. For further mass spectrometry analysis, the supernatant was vacuum-dried and mixed with 100 μL of an acetonitrile aqueous solution (acetonitrile: water = 1:1, *v*/*v*); the solution was reconstituted, vortexed, and centrifuged at 14,000× *g* at 4 °C for 15 min, and 2 μL of the supernatant was injected for analysis. The samples were chromatographically separated with an ACQUITY UPLC BEH C18 column (100 mm × 2.1 mm, 1.7 μm, Waters, Milford, MA, USA). Electrospray ionization (ESI) in the positive and negative ion modes was used for detection. A Q-Exactive quadrupole-electrostatic field Orbitrap high-resolution mass spectrometer (Thermo Fisher Scientific, Waltham, MA, USA) was used for mass spectrometry analysis.

The SIMCA-P 14.1 software (Umetrics, Umea, Sweden) was used for pattern recognition. After the data were preprocessed through Pareto scaling, multidimensional statistical analysis was performed, including unsupervised principal component analysis (PCA), supervised partial least squares discriminant analysis (PLS-DA), and orthogonal partial least squares discriminant analysis (OPLS-DA). One-dimensional statistical analysis was performed including Student’s *t*-test and fold-of-variation analysis, and the R software was used to draw volcano plots.

### 2.5. Histology

Hematoxylin-and-eosin (H&E) staining was performed to visualize the overall thoracic aorta structure and morphology. Collagen was stained using a Masson’s trichrome reagent to detect fibrosis, and Elastica van Gieson was used to detect elastin. The quantitation of collagen and elastin in the aorta was carried out by proportioning the collagen or elastin field area to the total area. The transmural length of the aortic media was measured on the same eight points of the aortic transection in all the aortas, and the average of eight measurements was presented as the medial thickness of the aorta.

### 2.6. Dihydroethidium (DHE) Staining

The frozen sections of the thoracic aorta tissue were washed with PBS and incubated with DHE in the dark for 1 h at room temperature. Red fluorescent signal was visualized by microscope (BX55, Olympus, Tokyo, Japan), and the number of red fluorescent dots was counted using ImageJ.

### 2.7. Immunohistochemistry

The thoracic aorta sections were stained with an immunohistochemistry kit (SP0041, Solarbio, Beijing, China). Briefly, antigen retrieval was conducted by immersing the samples in a citrate–EDTA buffer and then placing them in a microwave oven for 15 min at low-to-moderate power. Non-specific staining was blocked by using a 10% goat serum. After blocking, 50 μL of each of the diluted primary antibodies (CD45, 70257S, Cell Signaling Technology, Danvers, MA, USA; calponin, 380830, Zen Bioscience, Durham, NC, USA; α-smooth muscle actin, 14395-1-AP, Proteintech, Rosemont, IL, USA; vimentin, WL01960, Wanleibio, Shenyang, China; osteopontin, 340690, Zen Bioscience) was applied onto each section overnight at 4 °C. After incubation with a secondary antibody, the sections were visualized after a 2 min exposure to diaminobenzidine (DAB). The sections were then counter-stained with Myer’s hematoxylin for 2 min and then dehydrated and mounted with DePex.

### 2.8. Measurement of Leucine Level in Plasma and Aortas

Aortic lysate or plasma samples were obtained from the mice in different groups (10 mg/sample). The levels of leucine were analyzed by using an ELISA kit (FT-P9S2847X, Fantaibio, Shanghai, China) following the manufacturer’s protocol. The absorbance was measured at 450 nm on a microplate spectrophotometer (GENios Plus, Tecan, Switzerland).

### 2.9. Foxo1 Transcription Factor Assay

The nuclear extracts were obtained from the aortic tissue. A Foxo1 transcription factor activity assay was conducted by using an ELISA kit (ab207204, Abcam, Cambridge, UK) following the manufacturer’s protocol, and the absorbance was measured at OD450 nm on a microplate spectrophotometer (GENios Plus, Tecan, Switzerland).

### 2.10. Western Blots

The total protein was extracted from the aortic tissues by using a RIPA buffer and protease inhibitors. Proteins (30 µg) were resolved using 12.5% SDS–PAGE and then transferred to a polyvinylidene difluoride (PVDF) membrane with a Bio-Rad Western blotting system. The membrane was incubated with primary antibodies, including Slc3a2 (PA596401, Thermo Fisher), Slc7a5 (11326-1-AP, Proteintech), Sirt1 (ab189494, Abcam), Foxo1 (WL02491j, Wanleibio), Acetyl-Foxo1 (Lys294) (AF2305, Affinity Bioscience, Cincinnati, OH, USA) at 4 °C overnight and then with horseradish peroxidase-conjugated secondary antibodies (1: 2500) for 1 h at room temperature. All blots were developed by using a chemiluminescence system, and the signal intensities were analyzed with a Gel-Pro 4.5 Analyzer (Media Cybernetics, Rockville, MD, USA). The membranes were re-probed with β-actin to verify the loading consistency and normalize the target proteins.

### 2.11. Quantitative Reverse Transcription–Polymerase Chain Reaction (qRT-PCR)

The total RNA was extracted from mouse aortas using a TRIzol reagent. cDNA was synthesized using a cDNA reverse transcription kit (RR047A, Takara, Shiga, Japan) following the manufacturer’s protocol. qRT-PCR reactions were performed on a 7500 fast system (Thermos Fisher Scientific, USA). The relative levels of target genes were determined using the 2^−^^△△CT^ method. NOX4 primers: 5′-CCGAACACTCTTGGCTTACCTCC-3′, 5′-AGCAGCCCTCC TGAAACATGCAA-3′; β-actin primers: 5′-CATTGCTGACAGGATGCAG AAGG-3′, 5′-TGCTGGA AGGTGGACAGTGAGG-3′.

### 2.12. Statistical Analysis

All values are expressed as means ± SEMs. All the analyses were performed using Graphpad Prism 9. The significance of the differences between the two groups was assessed using a *t*-test, whereas one-way analysis of variance (ANOVA) was conducted for multiple-group comparisons. *p* < 0.05 was considered statistically significant.

## 3. Results

### 3.1. Aging Impaired Vascular Function and Promoted Vascular Remodeling

To establish the impacts of aging on vascular function, blood pressure and ex vivo vascular relaxation assays were conducted. SBP was comparable between the young and aging groups (Supplementary Figure S1A). Ach-mediated endothelium-dependent vasodilation was markedly impaired in the aging group (Supplementary Figure S1B). Meanwhile, the endothelium-independent vasodilation at the concentrations of 10^−8^–10^−6^ mol/L (SNP) was also impaired in the aged mice (Supplementary Figure S1C). Additionally, we assessed vascular morphology using H&E staining. Compared with the young mice, the aortas of the aged mice had a 37.3% increase in lumen diameter and 54.5% increase in wall thickness, but the ratio of the wall thickness to the lumen diameter was comparable between the two groups (Supplementary Figure S1D–G).

### 3.2. Metabolic Profile of Aging Aortas

To identify the aging-related metabolites in the aortas, liquid chromatography–tandem mass spectrometry was used to profile the metabolism characteristics of the young and aged aortas. Among the 196 metabolites identified, 104 metabolites were significantly different between the young and aged aortas, 71 were increased (red dots), and 33 were decreased (blue dots) (Supplementary Figure S2A). The changed metabolites were displayed in a heat map (Supplementary Figure S2B). Among these metabolites, the fold change in L-leucine ranked second. Compared with the aortas of the young mice, L-leucine was reduced in the aortas of the aged mice.

### 3.3. Leucine Supplementation in Middle-Aged Mice Improved Aging-Induced Vascular Dysfunction and Remodeling

**Figure 1:**
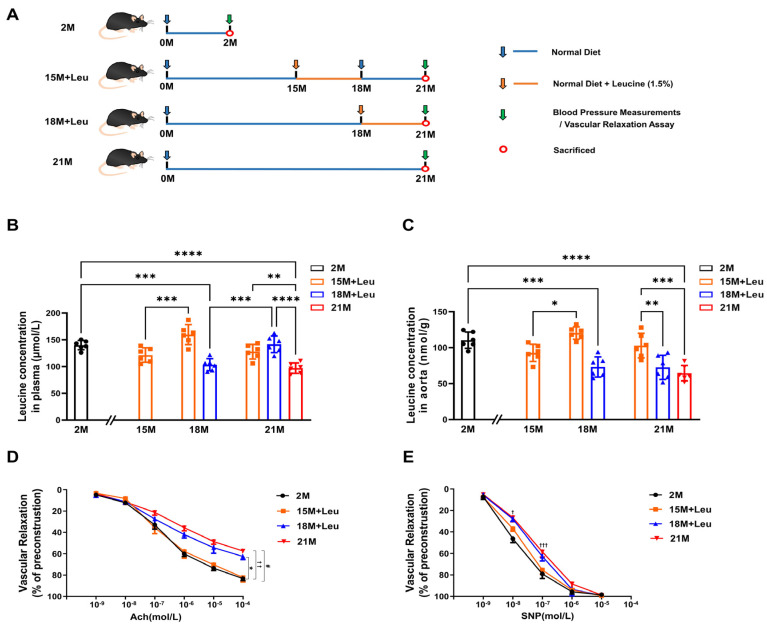
**Leucine supplementation from 15M alleviated aging-induced vascular dysfunction.** The design of study is shown in (**A**). Measurements of leucine levels in plasma and aorta are shown in (**B**,**C**). Acetylcholine-mediated endothelium-dependent relaxation and SNP-mediated endothelium-independent relaxation are presented in (**D**,**E**). Statistical analysis of leucine measurements was performed using one-way ANOVA, * *p* < 0.05, ** *p* < 0.01, *** *p* < 0.001, **** *p* < 0.0001. Statistical analysis of vascular relaxation curves was performed using two-way ANOVA, * *p* < 0.05 18M + Leu vs. 2M group, ^†^ *p* < 0.05 21M vs. 2M group, ^††^ *p* < 0.01 21M vs. 2M group, ^†††^ *p* < 0.001 21M vs. 2M group, ^#^ *p* < 0.05 18M + Leu vs. 15M + Leu group. All data are presented as mean ± SEM, *n* = 6/group.

Due to the reduction in leucine in the aged aortas, we hypothesized that leucine supplementation could ameliorate aging-induced vascular dysfunction and remodeling. We chose two time points, 15 months and 18 months, as the initial timing of leucine supplementation, and treatment duration was 3 months (Figure 1A). The plasma level of leucine in the aged mice (18 months old and 21 months old) was significantly reduced but not in the mice at the age of 15 months. After the 3-month supplementation, plasma leucine was significantly increased in the mice from both the 15M + Leu and 18 + Leu groups (Figure 1B). Consistent with these results, we also found that leucine in the aorta decreased with age. The leucine supplementation starting from 15 months reversed the decline in the aortic leucine levels in the aged mice; such an effect was not observed in the mice from the 18 + Leu group (Figure 1C). Body weight and food intake were measured in the different groups from 15 to 21 months. We found that the 15M + Leu group had lower weight gain than the 18M + Leu and 21M groups during the 15 to 18 months under study (Supplementary Figure S3A). The food intake was comparable among the four groups (Supplementary Figure S3B). No animal died during the study period. Leucine supplementation had no effect on SBP (Supplementary Figure S3C). Leucine supplementation starting from 15 months attenuated the aging-induced endothelium-dependent and -independent vasodilation, while these benefits were not observed in the mice with leucine supplementation starting from 18 months (Figure 1D,E).

**Figure 2:**
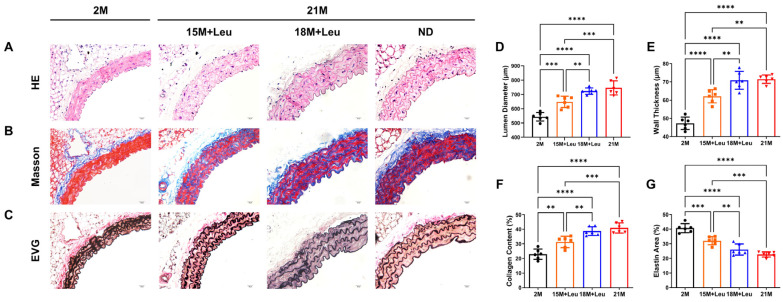
**Leucine supplementation from 15M improved aging-induced vascular remodeling.** Representative images of H&E (**A**), Masson’s (**B**), and van Gieson’s (**C**) staining show the aortic structure of different groups at low and high magnifications. Lumen diameter and wall thickness of each group were measured (**D**,**E**). The percentage of collagen deposition area is shown in (**F**). Quantification of elastin area is shown in (**G**). Statistical analysis was performed using one-way ANOVA; data are presented as mean ± SEM, *n* = 6/group. ** *p* < 0.01, *** *p* < 0.001, and **** *p* < 0.0001. ND: normal diet.

Vascular remodeling was determined using H&E, Masson’s Trichrome, and van Gieson’s staining. Compared with the aged group without leucine supplementation, the aortas from the 15M + Leu group had a 15.1% reduction in the lumen diameter and a 14% decrease in the medial layer thickness (Figure 2A,D,E). Masson’s Trichrome staining revealed collagen content in the medial layer was significantly increased in the aged group, whereas leucine supplementation from 15 months reduced aging-induced collagen deposition (Figure 2B,F). Additionally, the mice with leucine supplementation starting from 15 months ameliorated the elastin loss and promoted the normalization of vascular structure (Figure 2C,G). By contrast, these improvements were not observed in the 18M + Leu group.

### 3.4. Leucine Supplementation Relieved Aging-Induced Vascular Inflammatory Responses, ROS Generation, and VSMC Phenotype Transformation

**Figure 3:**
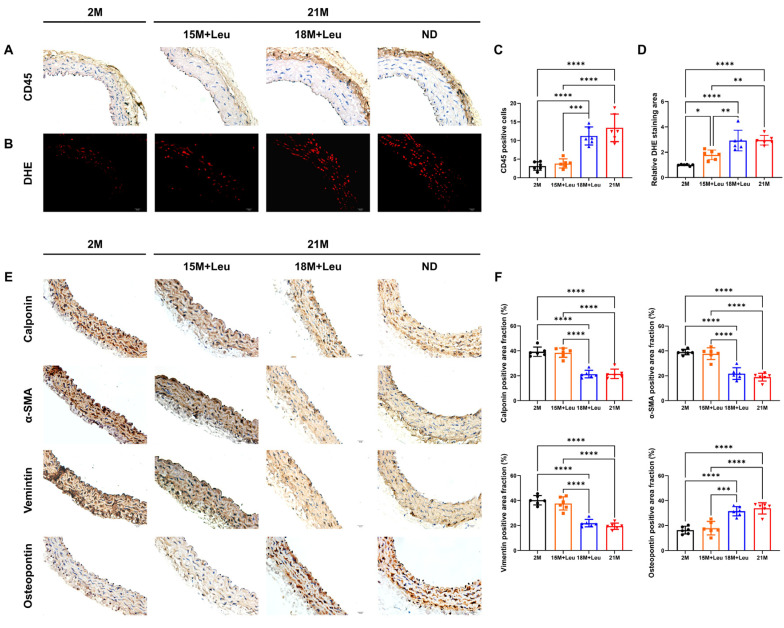
Leucine supplementation from 15M relieved aging-induced vascular inflammatory responses, ROS generation, and VSMC phenotype transformation. Representative images of CD45 immunohistochemistry staining showed inflammatory cell infiltration (**A**). Representative images of DHE staining (**B**). Quantitative analysis of CD45 positive cells is shown in (**C**). Quantitative analysis of the red dots is shown in (**D**). Representative immunohistochemistry staining and quantification of contractile markers calponin, ɑ-SMA, vimentin, and synthetic marker osteopontin are shown in (**E**,**F**). Statistical analysis was performed using one-way ANOVA; data are presented as mean ± SEM, n = 6/group. * *p* <0.05, ** *p* < 0.01, *** *p* < 0.001, and **** *p* < 0.0001. ND: normal diet.

Aging significantly increased inflammatory cell infiltration and vascular ROS levels, as indicated by immunohistochemistry (CD45) and DHE staining, which were abolished with leucine supplementation starting from 15 months (Figure 3A–D). This benefit was not observed in the mice with leucine supplementation at 18 months.

The VSMCs in the aged mice displayed a transdifferentiation state, as evidenced by a reduction in VSMC contractile markers, calponin, ɑ-SMA, and vimentin, and an increase in the synthetic marker osteopontin. Leucine supplementation starting from 15 months prevented VSMC phenotype transformation, as evidenced by the repression of the decline in contractile markers and the increment in synthetic markers in the aged aortas (Figure 3E,F). These benefits were not observed in the mice with leucine supplementation from 18 months.

### 3.5. Leucine Protected against Aging-Induced Vascular Remodeling via Sirt1–Foxo1 Pathway

**Figure 4:**
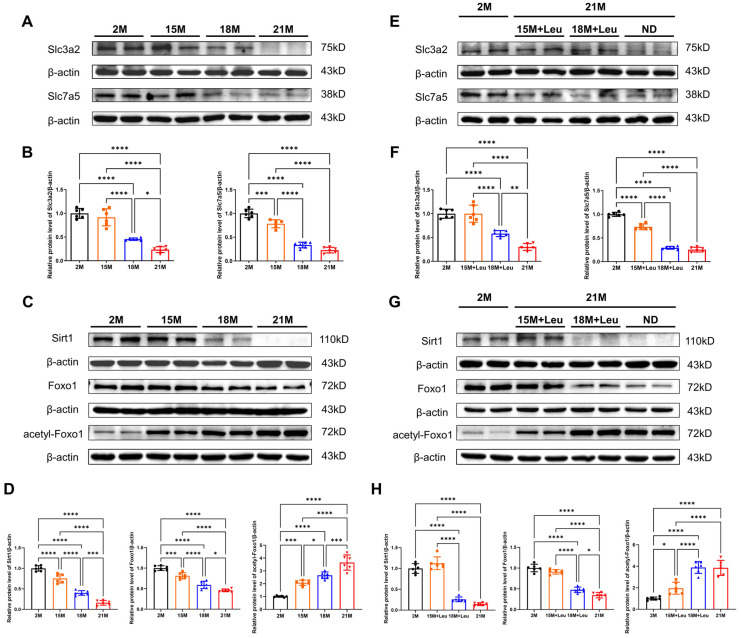
**Leucine protected against aging-induced vascular damage via Sirt1–Foxo1 pathway.** Representative Western blot and quantification of vascular leucine transporters Slc3a2 and Slc7a5 from mice at different ages are in (**A**,**B**). Representative Western blot and quantification of Sirt1, Foxo1, and acetyl-Foxo1 from mice at different ages are shown in (**C**,**D**). Representative Western blot and quantification of vascular Slc3a2 and Slc7a5 from mice in different intervention groups are shown in (**E**,**F**). Representative Western blot and quantification of Sirt1, Foxo1, and acetyl-Foxo1 from mice in different intervention groups are shown in (**G**,**H**). Statistical analysis was performed using one-way ANOVA; data are presented as mean ± SEM, n = 6/group. * *p* < 0.05, ** *p* < 0.01, *** *p* < 0.001, and **** *p* < 0.0001. ND: Normal diet.

The major leucine transporters Slc3a2 and Slc7a5 were slightly reduced in the aortas at 15 months but were dramatically reduced after 18 months, as indicated by our assessment of the mice at the ages of 2, 15, 18, and 21 months (Figure 4A,B). In parallel, a reduction in Sirt1 and an increase in acetyl-Foxo1 were observed (Figure 4C,D). The increase in acetyl-Foxo1 was associated with the repressed Foxo1 activity in the aorta of mice at different ages (Supplementary Figure S4A). The aging-induced reduction in Slc3a2 and Slc7a5 was restored with leucine supplementation, and such effects were more profound in the 15M + Leu group (Figure 4E,F). Meanwhile, the increase in acetyl-Foxo1 was repressed, and the decrease in Foxo1 activity was restored in the mice with leucine supplementation from 15 months (Figure 4G,H, Supplementary Figure S4B).

**Figure 5:**
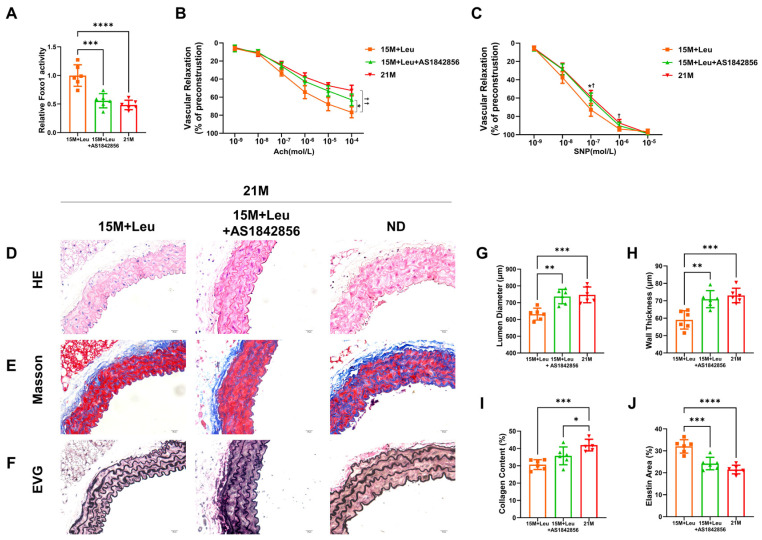
**Inhibition of Foxo1 activity reversed the protective effects of leucine supplementation on vascular dysfunction and remodeling**. Quantification of Foxo1 activation (**A**). Dose–response curves for acetylcholine-mediated endothelium-dependent relaxation (**B**) and SNP-mediated endothelium-independent relaxation (**C**). Representative images of H&E, Masson’s, and van Gieson’s in aortas from mice at 2M and 21M, and 21M mice with leucine supplementation from 15M, with or without Foxo1 inhibitor AS1842856 (**D**–**F**). Quantitative analysis is shown in (**G**–**J**). Statistical analysis of vascular relaxation curves was performed with two-way ANOVA, * *p* < 0.05 15M + Leu + AS1842856 vs. 2M group, ^†^ *p* < 0.05 21M vs. 2M group, ^††^ *p* < 0.01 21M vs. 2M group. Histological statistical analysis was performed with one-way ANOVA, * *p* < 0.05, ** *p* < 0.01, *** *p* < 0.001, and **** *p* < 0.0001. All data are presented as mean ± SEM, n = 6/group. ND: normal diet.

**Figure 6:**
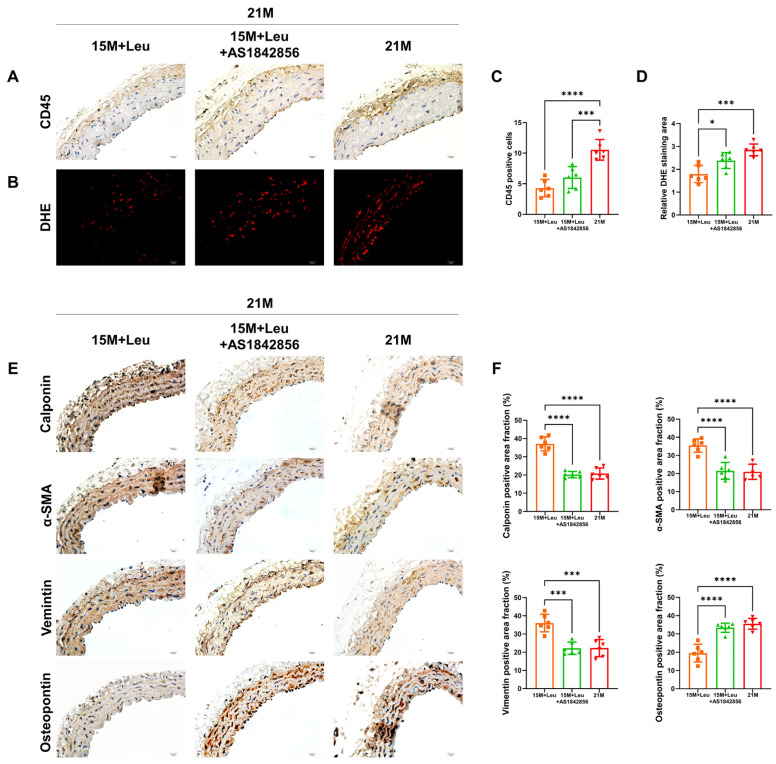
Inhibition of Foxo1 activity reversed the protective effects of leucine supplementation on VSMC phenotype transformation. Representative images and quantification of CD45 immunohistochemistry and DHE staining are shown in (**A–D**). Representative immunohistochemistry staining and quantification of contractile markers calponin, ɑ-SMA, vimentin, and synthetic marker osteopontin are shown in (**E**,**F**). Statistical analysis was performed using one-way ANOVA; data are presented as mean ± SEM, n = 6/group. * *p* < 0.05, *** *p* < 0.001, and **** *p* < 0.0001. ND: normal diet.

Next, we investigated whether the protective effects of leucine were via a Foxo1-dependent mechanism. The activity of Foxo1 in the aortas was inhibited after AS1842856 treatment (Figure 5A). The leucine-improved vascular dysfunction and remodeling were impaired in the mice with the Foxo1 inhibitor (Figure 5B–J). In addition, the Foxo1 inhibitor suppressed the leucine-maintained contractile phenotype of VSMCs (Figure 6E,F). The leucine-repressed ROS generation and inflammatory cell infiltration (Figure 6A–D) in the aged mice were only slightly reversed by the Foxo1 inhibitor.

## 4. Discussion

In the present study, for the first time, we revealed that vascular leucine was impaired during aging, which was mediated by the reduction in amino acid transporter Slc3a2 or Slc7a5. Dietary leucine supplementation at the middle-age stage (15 months), not the late stage (18 months), maintained the contractile phenotype of VSMCs and restrained the vascular inflammatory responses and ROS generation in the aged mice, partially via activating the Sirt1-induced Foxo1 deacetylation, which may shed light on therapeutic strategies for aging-induced vascular remodeling and dysfunction.

Alterations in metabolism are a common feature of cardiovascular diseases, with most studies focused on fatty acid [8] and glucose utilization [9]. However, little is known about the changes in amino acid concentration and their potential functions in the pathogenesis of cardiovascular diseases. Amino acids modulate cell function not only as nutrients but also as contributors to the regulation of signaling pathways [10]. Branched-chain amino acids (BCAAs), including leucine, isoleucine, and valine, are essential amino acids with a shared catabolic pathway, which are obtained from high-protein foods [19,20]. Several clinical studies reported that aging was associated with decreased plasma levels of BCAAs [21,22]. Consistent with these studies, in the present study, leucine was markedly reduced in the aortas of aged mice. One of the possible mechanisms for the leucine deficiency was likely due to less dietary protein intake in the aged population [23]. Additionally, aging impaired BCAA biosynthesis and metabolism [10]. In old rats, leucine synthesis in skeletal muscle was lower than that in adults [24]. The islets from 12-month-old rats manifested a lower response to leucine stimulation, resulting in reduced insulin secretion as compared to the islets from 2-month-old rats [25]. Decreased mitochondrial DNA copy numbers were found in the skeletal muscle of aged rats, coupled with an aging-induced decline in muscle mass, which led to the reduced capacity to catabolize BCAAs [19,26].

Currently, the principal treatment for BCAA deficiency is to restore blood BCAA supply, which can be achieved by dietary supplementation. Both clinical and experimental studies reported that dietary supplementation with BCAA exerted beneficial effects on age-related diseases. Leucine supplementation was able to promote skeletal muscle protein synthesis and was recommended as an effective therapeutic approach for curing elderly sarcopenia patients [27,28]. Mansfeld et al. demonstrated that supplementation of BCAAs extended the lifespan of nematodes, zebrafish, and mice [29]. D’Antona et al. showed that BCAA supplementation increased mitochondrial biogenesis and function and reduced ROS production in the cardiac and skeletal muscles of middle-aged mice [30]. Nevertheless, little is known about the effectiveness of leucine supplementation in aged vessels. In the present study, we found that leucine supplementation starting from 15 months improved vascular remodeling and dysfunction, by maintaining the contractile phenotype of VSMCs, thus suppressing inflammatory cell infiltration and ROS generation.

However, the effects of BCAA supplementation on aging remain controversial. Solon-Biet et al. found that long-term exposure to high BCAA diets led to hyperphagia, obesity, and reduced lifespan in mice [31]. Lin et al. found that, compared with age >75 years-old patients, only relatively younger sarcopenic patients (65–74 years old) had a better physical performance from leucine supplementation [32]. Therefore, the dosage, duration, and timing of administration were regarded as critical issues for its efficacy. In this study, we selected a low dose of leucine (1.5%) for a 3-month duration as the treatment regimen and tested two times of administration, 15 months or 18 months. Surprisingly, with the same dosage and duration, improvements in age-related vascular dysfunction and remodeling were only observed in the mice with leucine supplementation starting from 15 months but not from 18 months. Amino acid uptake is dependent on transporters; therefore, we speculated that a different level of efficacy may attribute to alterations in leucine transporters at different ages. Slc3a2 and Slc7a5 are L-type amino acid transporters and are mainly responsible for leucine uptake [33]. A decreased expression of Slc3a2 was found in aged skin and senescent dermal fibroblasts [34,35]. Chronic exercise training or leucine supplementation can upregulate the expression of Slc3a2 and Slc7a5 [36,37]. In the current study, we found a significant reduction in vascular Slc3a2 and Slc7a5 occurred at the age of 18 months, which explained the lack of efficacy of leucine supplementation from 18 months. Consistent with our results, Dickinson et al. reported that the supplementation of essential amino acids failed to increase the content of Slc3a2 and Slc7a5 in older people, compared with young individuals [38]. Interestingly, leucine supplementation from 15 months completely reversed the reduction in Slc3a2 and Slc7a5 at 21 months, indicating that the stimulation of amino acid transporters at the middle-age stage prevented their downregulation.

Sirt1 was a NAD-dependent deacetylase, involved in the regulation of senescence through the acetylation/deacetylation of downstream substrates. Consistent with previous studies [39], our research showed the Sirt1 protein level was significantly suppressed in the aortas of the aged mice. Additionally, we found that leucine supplementation reversed the aortic Sirt1. Foxo1, belonging to a family of transcriptional regulators characterized by a conserved DNA-binding domain termed the forkhead box, is one of the Sirt1 targets and is activated by Sirt1-mediated deacetylation. The loss of Foxo1 transcriptional activity has been implicated in multiple vascular disorders. The inactivation of Foxo1 led to an increase in cyclins (cyclin B1 and cyclin D1) and a decrease in CDKN1B (p27), which may contribute to the hyperproliferation of vascular cells [40]. Lu et al. found that the downregulation of Foxo1 promoted the inflammatory responses of VSMCs in db/db mice [41]. Moreover, Norambuena et al. showed that Foxo1 silencing blocked the preventive effects of angiotensin-(1–9) on platelet-derived growth factor-BB (PDGF-BB), thus inducing VSMC synthetic phenotype transition [42]. Here, we provided strong evidence that a reduction in Sirt1 was associated with the aging-induced VSMC synthetic phenotype transition. Both a reduction in Sirt1 and VSMC synthetic phenotype transition were reversed by leucine supplementation. We further confirmed that Sirt1-mediated Foxo1 deacetylation was essential for maintaining the VSMC contractile phenotype, highlighting the importance of Foxo1 activity in the aging-induced VSMC phenotype transition. Of interest was the finding that the leucine-supplementation-reversed VSMC phenotypic transition in the aging mice was inhibited by the Foxo1 inhibitor, but the leucine-repressed ROS generation and vascular inflammatory responses were only weakly suppressed, suggesting that the impaired inflammatory responses and ROS by leucine supplementation were not fully dependent on Foxo1; other Foxo1-independent mechanisms might be involved, which needs further investigation.

Amino acid transporters Slc3a2 and Slc7a5 are also used for transporting other amino acids, such as aspartic acid or glutamine. The reduction in Slc3a2 and Slc7a5 may indicate that aortic aspartic acid or glutamine is also reduced. Whether a reduction in other amino acids contributes to aging-induced vascular remodeling is unknown. Aging is associated with mitochondrial injury, which subsequently leads to metabolic remodeling [43]. On the other hand, within the cell, leucine is eventually converted to acetoacetate acid and acetyl-CoA, which are the intermediates of the tricarboxylic acid cycle [44]. Therefore, the aortic deficiency of leucine may further accelerate mitochondrial injury [44]. Mitochondrial injury causes excessive ROS generation, inflammatory responses, and VSMCs. Whether mitochondrion-targeted antioxidant therapy such as Mito-TEMPO can reverse the aging-induced reduction in Slc3a2 and Slc7a5, as well as aortic deficiency, needs further study.

Notably, aortic phenylalanine is increased in aged mice. Both leucine and phenylalanine are essential amino acids, which are obtained from the diet; thus, changes in leucine and phenylalanine were expected to move in the same direction. However, leucine and phenylalanine displayed distinct changes in the aged aortas. Diet is not the only mechanism for regulating plasma or regional amino acid metabolism. The catabolism of leucine and phenylalanine is controlled by different enzymes. Phenylalanine is regulated by the tetrahydrobiopterin-dependent rate-limiting enzyme phenylalanine hydroxylase (PAH), the expression of which is physiologically restricted to the liver and kidneys [45]. Czibik et al. indicated that there was a markedly reduced hepatic PAH protein level in old wild-type mice. Reduced phenylalanine catabolism caused an increased level of phenylalanine in the plasma and subsequently accumulated in the heart. Phenylalanine is detrimental to the aged heart, and the restriction of phenylalanine in the diet is beneficial to the aged heart [46]. Whether the restriction of phenylalanine in the diet is beneficial to the aged aorta deserves further investigation.

## 5. Limitations

We had several limitations in the current study. *First*, we only used male mice for the whole study. In adult female mice, estrogen has a protective effect on cardiovascular diseases. Estrogen is markedly reduced in the aged mice, whether the aged female mice have similar effects to leucine needs further study. *Second*, we used the thoracic aorta for this study. Whether the ascending aorta and abdominal aorta undergo aging-induced remodeling or have similar responses to leucine is still unknown. *Third*, this study was terminated at 21 months. How long the effects of leucine treatment can last is still unknown.

## 6. Conclusions

**Figure 7:**
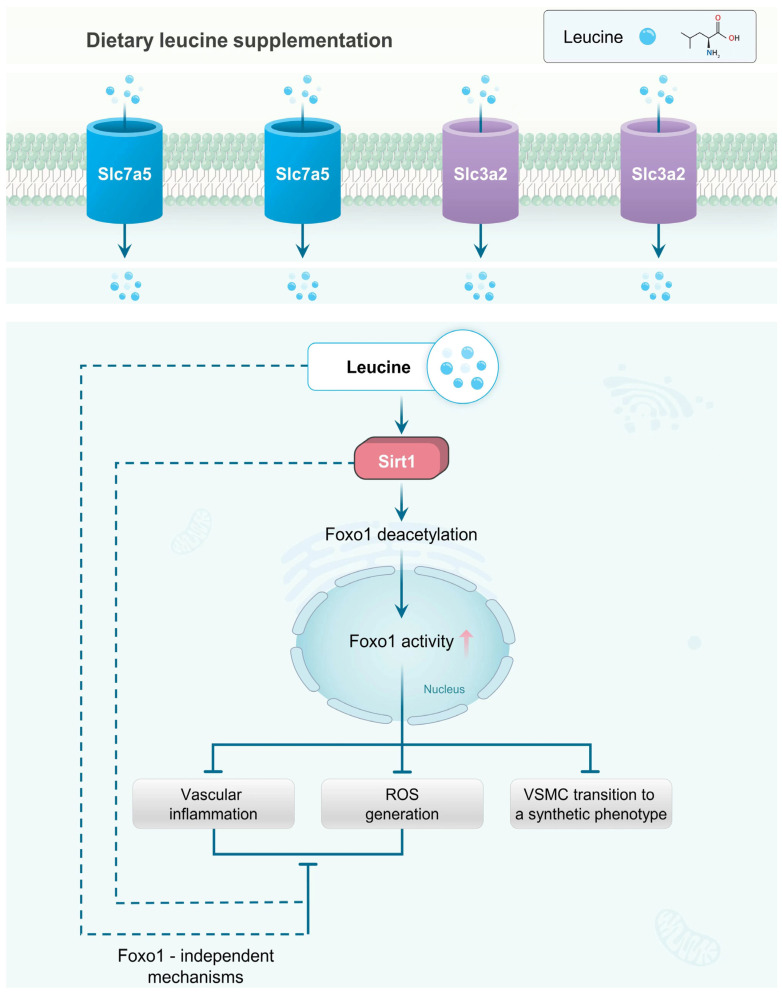
**Mechanisms of leucine in the protection of aging-induced vascular remodeling.** Transport of leucine depends on amino acid transporters Slc7a5 and Slc3a2. Intracellular leucine increases protein level of Sirt1, which subsequently induces Sirt1-mediated Foxo1 deacetylation. Foxo1 deacetylation enhances Foxo1 activity in nucleus, which represses VSMC transition to a synthetic phenotype, vascular inflammatory responses, and excessive ROS generation. Meanwhile, leucine or Sirt1 may also relieve vascular inflammation and ROS level via Foxo1-independent mechanisms. Dietary leucine supplementation at middle-age stage reversed aging-induced vascular remodeling and dysfunction.

Aging-induced vascular remodeling and dysfunction can be reversed by dietary leucine supplementation at the middle-age stage by repressing the VSMC transition to a synthetic phenotype, vascular inflammatory responses, and ROS generation in aged mice. These benefits are in part via activating the Sirt1-induced Foxo1 deacetylation. Meanwhile, leucine or Sirt1 may also relieve aging-induced vascular inflammation and ROS levels via Foxo1-independent mechanisms (Figure 7).

## Data Availability

The data presented in this study are available on request from the corresponding authors.

## Supplementary Materials

The following supporting information can be downloaded at: https://www.mdpi.com/article/10.3390/nu14183856/s1, Figure S1: Aging impaired vascular relaxation and promoted aortic wall thickening and lumen enlargement. Figure S2: Metabolic profiling of aortas from mice at the age of 2M and 21M. Figure S3: Leucine supplementation from 15 months reduced the body weight. Figure S4: Leucine supplementation from 15 months reversed aging-induced Foxo1 activity decline.
