# Supplementary material for: Leucine Supplementation in Middle-Aged Male Mice Improved Aging-Induced Vascular Remodeling and Dysfunction via Activating the Sirt1-Foxo1 Axis
Source: Nutrients. 2022 Sep 17;14(18):3856. doi: 10.3390/nu14183856 (PMC9505861; doi:10.3390/nu14183856)

Supplemental Material

Leucine Supplementation in Middle-Aged Male Mice Improved Aging-Induced Vascular Remodeling and Dysfunction via Activating Sirt1-Foxo1 Axis

Zhujing Hao[1#], Guiwen Xu[1#], Mengyang Yuan[1], Ruopeng Tan[1], Yun-Long Xia[1], Yang Liu[1*], Xiaomeng Yin[2*]

[1]Institute of Cardiovascular Diseases, the First Affiliated Hospital of Dalian Medical University, Dalian, China; [2]Department of Cardiology, the First Affiliated Hospital of Dalian Medical University, Dalian, China

# These authors contributed equally to this work.

Correspondence*: Xiaomeng Yin, Department of Cardiology, First Affiliated Hospital of Dalian Medical University, No 222 Zhongshan Rd, Dalian, China; E-mail: dr.yinxm@163.com and Yang Liu, Institute of Cardiovascular Diseases, the First Affiliated Hospital of Dalian Medical University, No 222 Zhongshan Rd, Dalian, China. Email: liuyang19831119@163.com.

Supplemental Figure S1: Aging impaired vascular relaxation and promoted aortic wall thickening and lumen enlargement.

SBP measurements by the noninvasive tail-cuff method in mice at the age of 2M and 21M **(A)**. Dose-response curves for acetylcholine mediated endothelium-dependent relaxation **(B)** and SNP mediated endothelium-independent relaxation **(C)**. Representative images of H&E staining of the aortas from mice at the age of 2M or 21M **(D)**. Lumen diameter and wall thickness of each group were measured **(E-F)**, and the ratio of wall thickness and lumen diameter was calculated **(G)**. Statistical analysis of SBP measurements and H&E staining were performed by T-test, ****p<0.0001 vs 2M. Statistical analysis of vascular relaxation curves was performed by two-way ANOVA, ††p<0.01 21M vs 2M group, †††p<0.001 21M vs 2M group, ††††p<0.0001 21M vs 2M group. Data were presented as mean ± SEM, n = 6/group.

Supplemental Figure S2: Metabolic profiling of aortas from mice at the age of 2M and 21M.

Volcano plot revealed 104 metabolites with 2-folds change and p < 0.05 (2M/21M), red dots indicated increased metabolites, whereas blue dots indicated decreased **(A)**. A heat map of differential metabolites between 2M and 21M is shown in **(B)**. n = 4/group.

Supplemental Figure S3: Leucine supplementation from 15 months reduced the body weight.

Body weight and food intake was measured in different intervention groups from 15M to 21M, the quantification is shown in **(A and B)**. SBP measurements is shown in **(C)**. Statistical analysis was performed by one-way ANOVA, data were presented as mean ± SEM, n = 6/group. $^{\#}$p<0.05 18M+Leu or 21M vs 15M+Leu group.

Supplemental Figure S4: Leucine supplementation from 15 months reversed aging-induced Foxo1 activity decline.

Quantitative analysis of Foxo1 activity from mice at different ages is shown in (**A**). Quantitative analysis of Foxo1 activity from mice at different intervention groups is shown in **(B)**.

Supplemental Figure S1

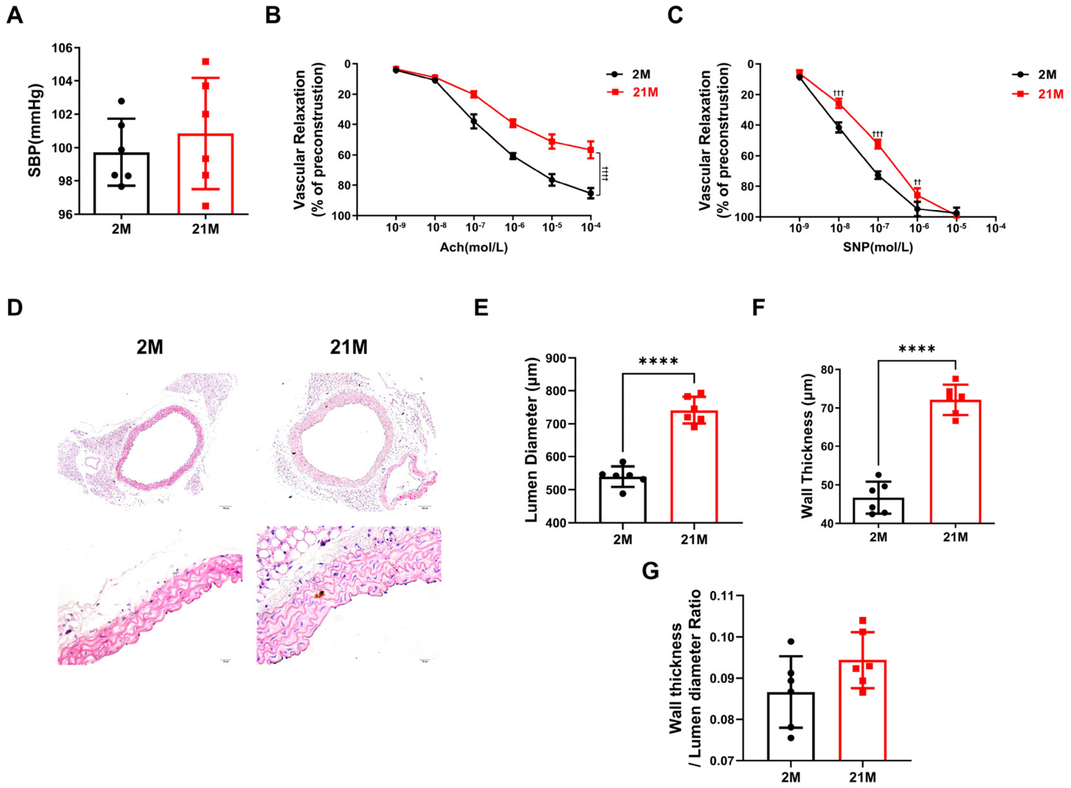

Supplemental Figure S2

A

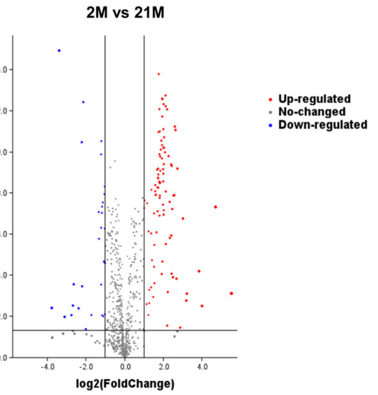

B

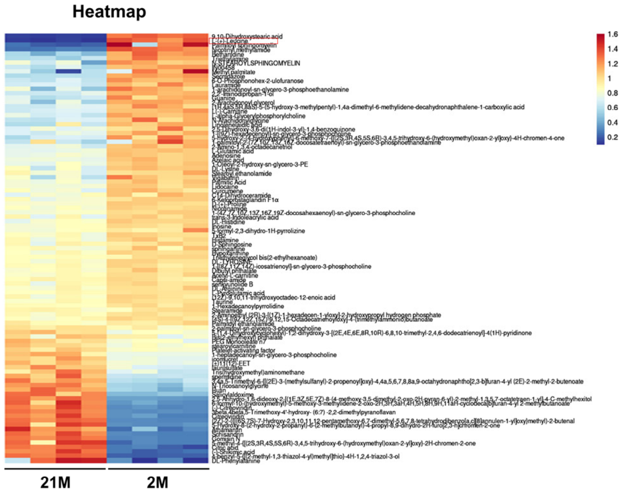

Supplemental Figure S3

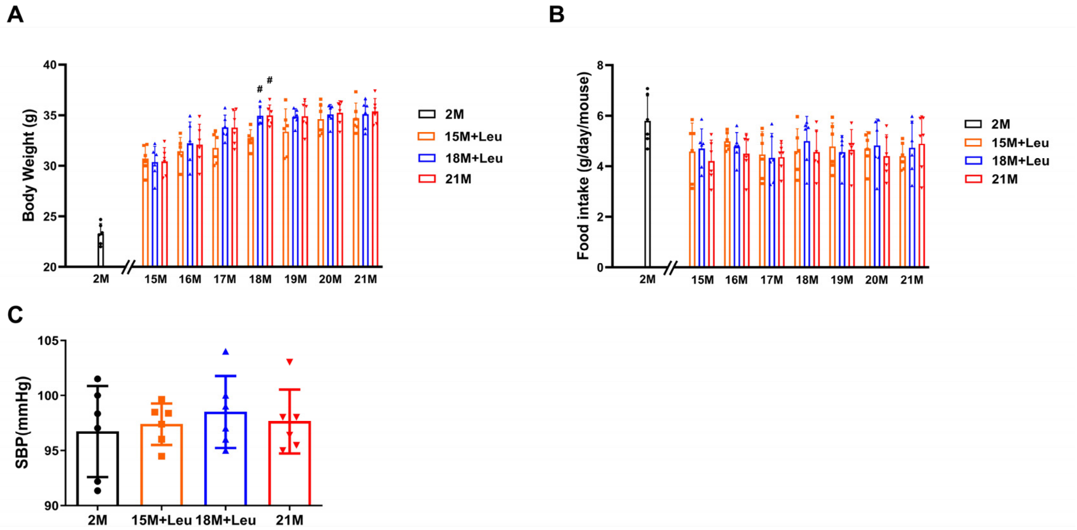

Supplemental Figure S4

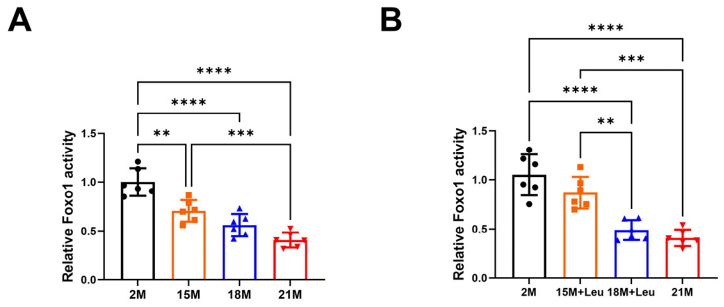

Supplement: Supplementary file 1 [file nutrients-14-03856-s001.zip › Supplemental Material.pdf]
