# Supplementary figures and images for: Leucine Supplementation in Middle-Aged Male Mice Improved Aging-Induced Vascular Remodeling and Dysfunction via Activating the Sirt1-Foxo1 Axis
Source: Nutrients. 2022 Sep 17;14(18):3856. doi: 10.3390/nu14183856 (PMC9505861; doi:10.3390/nu14183856)

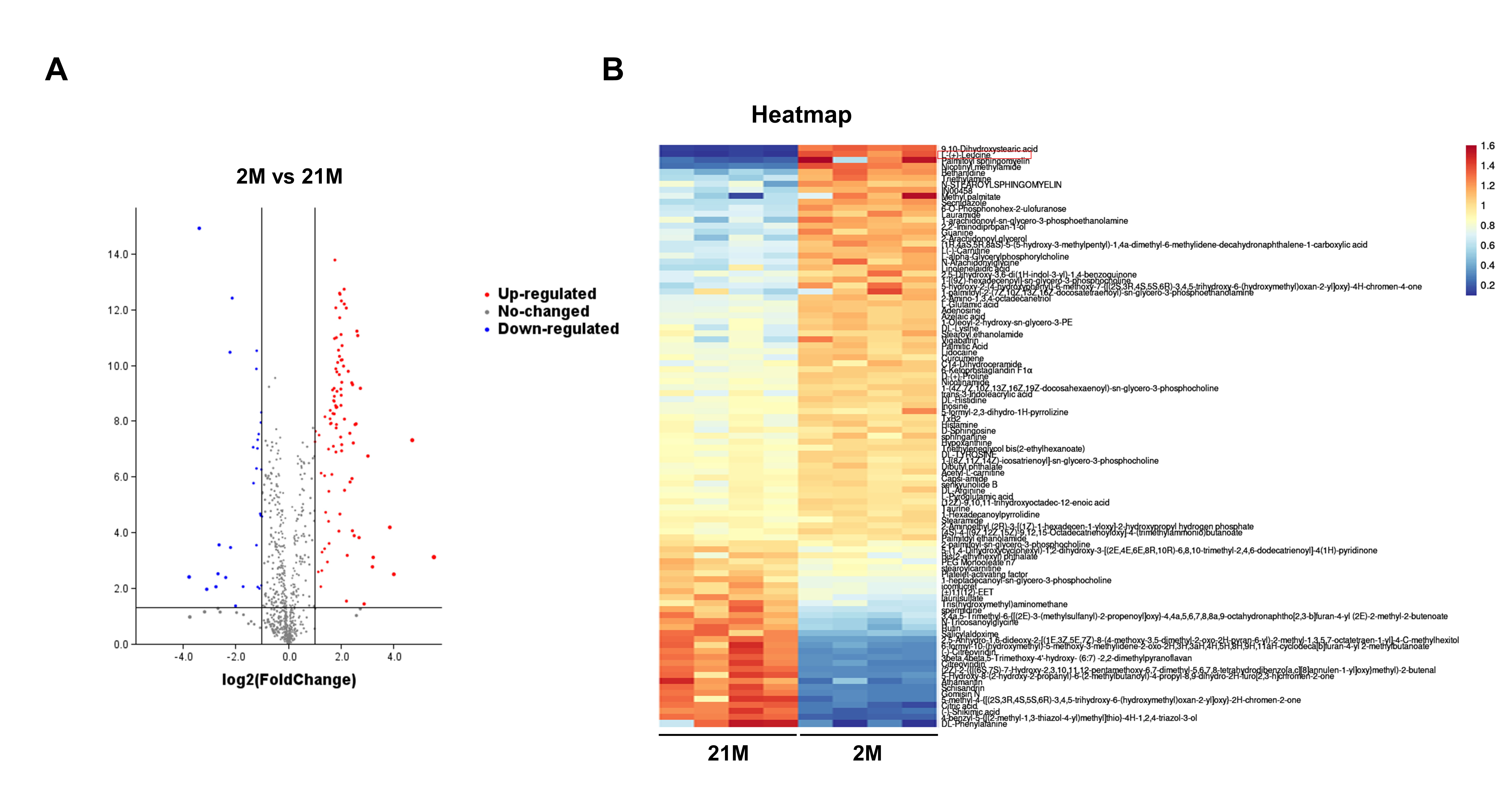

Supplement: Supplementary file 1 [file nutrients-14-03856-s001.zip › Supplemental Figures/Supplemental FigureS2.jpg]

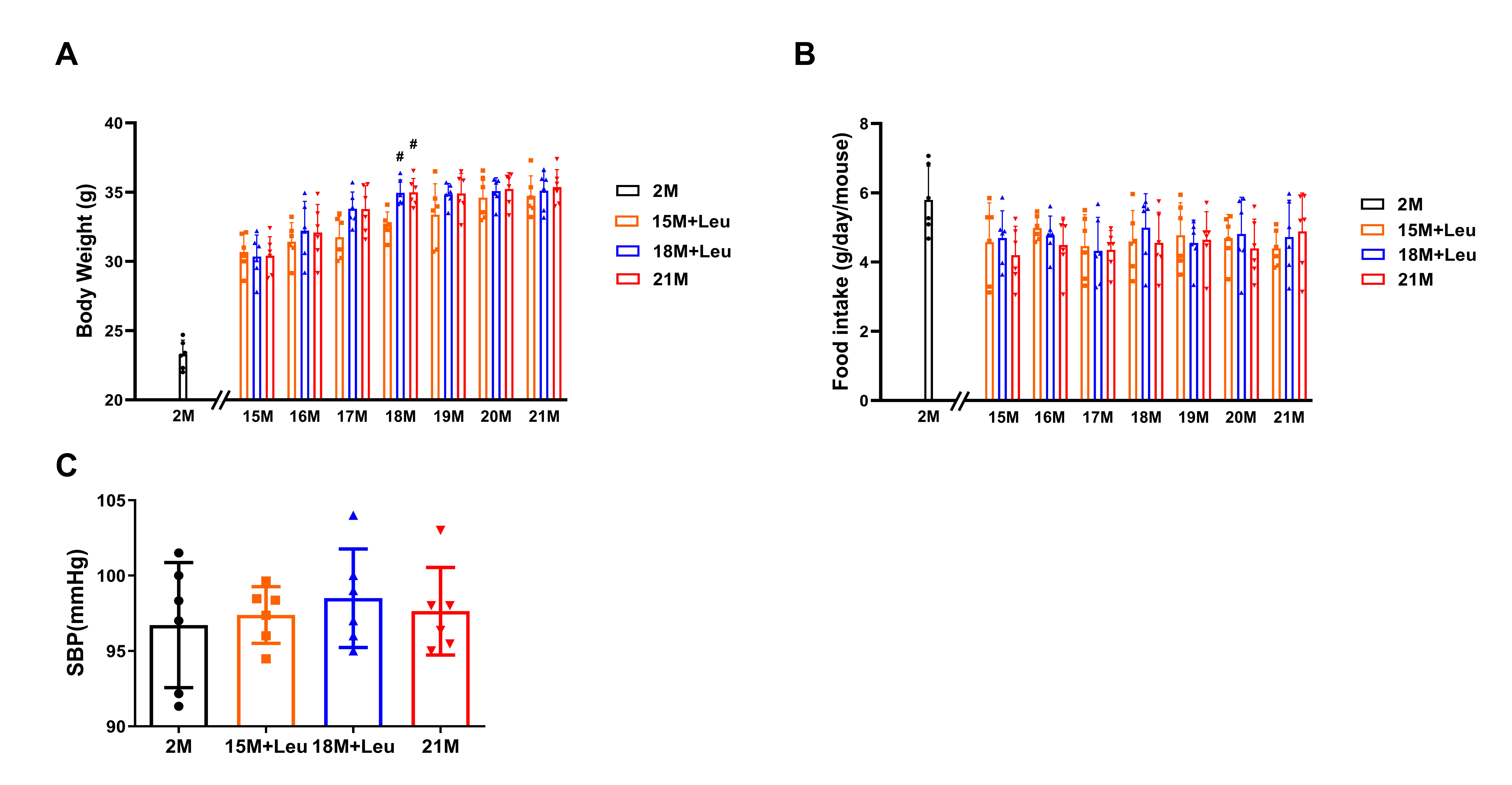

Supplement: Supplementary file 1 [file nutrients-14-03856-s001.zip › Supplemental Figures/Supplemental FigureS3.jpg]

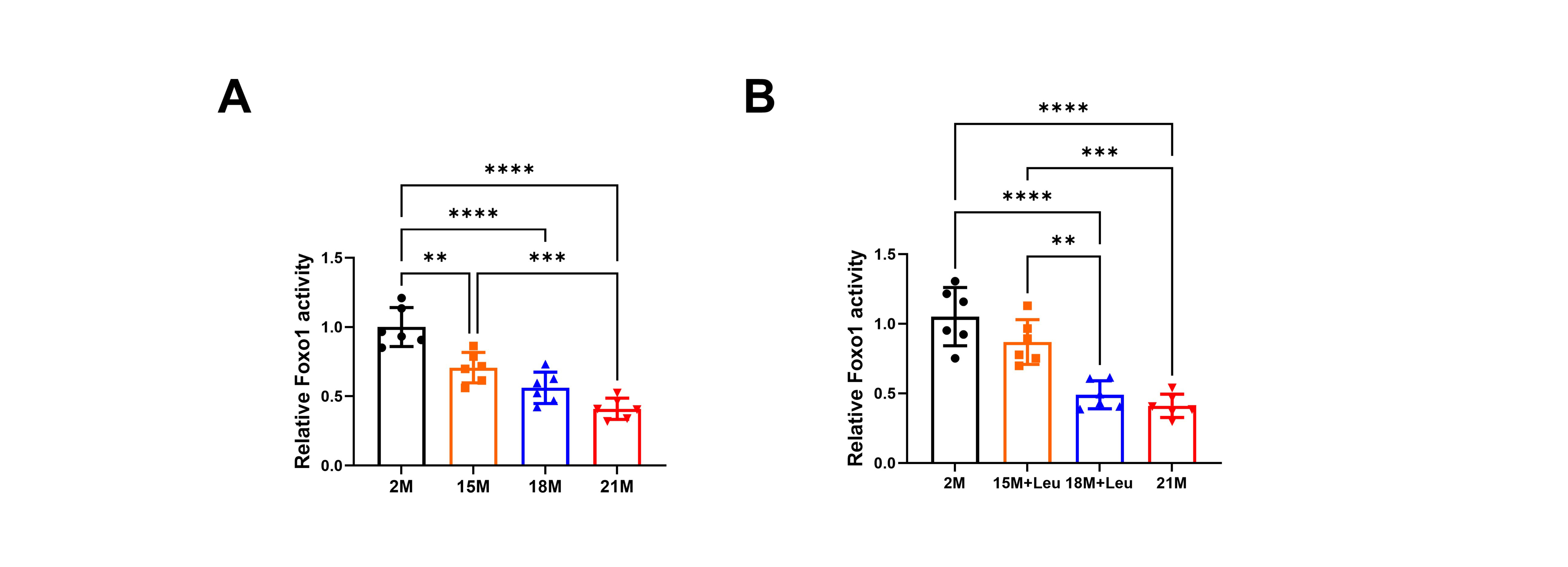

Supplement: Supplementary file 1 [file nutrients-14-03856-s001.zip › Supplemental Figures/Supplemental FigureS4.jpg]
